# Digital leadership role in developing business strategy suitable for digital transformation

**DOI:** 10.3389/fpsyg.2022.1066180

**Published:** 2023-01-04

**Authors:** Abdullah Türk

**Affiliations:** Department of Aviation Management, Organization, Istanbul Bilgi University, Istanbul, Turkey

**Keywords:** digital transformation, business strategy, organizational change, digital leadership, digital strategy

## Abstract

Businesses must respond to the ecology in which they operate. Especially the rapid transformation of technology has increased the degree of dependency on the system. The main reason for this is perceived only as the technology costs brought by digital transformation. However, we understand from the bankruptcy of economically strong companies that this is not the real problem. This study looks at it from the perspective of leadership, which is an important skill for businesses. The research focuses on leadership roles needed to adapt to digital transformation. At this point, the roles of digital leadership and its contribution to businesses were investigated. At this point, we try to reveal the role of digital leadership with two different qualitative analyzes. In the research, semi-structured interviews were conducted with senior managers, phenomenological and content analysis was performed using Nvivo and MAXQDA qualitative analysis programs, and relevant confidential information was revealed. As a result of the research, it has been determined that there is an important link between time management and productivity while supporting system efficiency and transformation adaptation. In other words, a positive relationship has been determined between the success of digital transformation and digital leadership roles. In digital transformation, digital leadership has a role in the positive development of the relationship between the digital transformation process and business strategies. As a result, a perspective on how digital leadership can contribute to businesses that want to develop strategies suitable for the digital transformation process is presented.

## Introduction

Increasing dynamics in the corporate environment shaped by digital transformation with Industry 4.0 lead to difficulties that create constant complexity in the management of companies. Being a follower of every short-term or long-term move is now vital to surviving in this dynamic and complex corporate climate. The turnover rate of technology in digital transformation also tests the tactical action capacity of businesses on the way to strategic goals. Therefore, the capacity of an enterprise to catch up-to-date mobility becomes an important criterion in the success of the business strategy. In a way, this can be thought of as the capacity to keep up with the current development. Anghel (2019) took this situation one step further and interpreted compliance in digital transformation as purchasing the change process of leaders, managers, employees and integrating the organization’s systems with new digital technologies. The most important point here is to act with foresight and not only adapt to innovation, but also to be the pioneer of innovation, to manage time effectively as a demander of innovation. At this point, our motto is “Waking up early goes forward.” It is necessary to correctly understand the relationship between business strategy and the leader. Because the technological returns of each period bring new paradigms to the fore. There are some concepts that need to be known in order to understand the paradigm in the new age. These concepts are listed below. Digital transformation is the strategic adoption of new digital technologies and tools to improve business processes and productivity, as well as deliver better customer service and employee experiences (Citrix Systems, Inc., 2020). The digital strategy is; effective rethinking of business processes, customer relations and management practices that provide competitive advantage and value creation (Wald et al., 2019). The most important feature required for this is the digital mindset. Digital mindset; It includes the demonstration of features and behaviors by members of the organization that maximizes business value from technology (Azzouzi, 2017). However, even if you have this mentality, it is also very important to be competent in this regard. Digital competence is; information management, collaboration, communication and sharing, content and knowledge creation, ethics and responsibility, evaluation and problem solving, and technical operations (Ferrari, 2012). Building a digital culture is necessary in order to spread and maintain the entire concept within the enterprise. Digital culture; It is formed by some characteristics of the culture of creation and cooperation culture. The culture of creation; It is a very dynamic and entrepreneurial structure that focuses on being the first to do something. People are willing to bet their ideas and take risks. The culture of cooperation is; they value the long-term development of the employee and support the evolution of people who integrate him. It is flexible and internal. Leaders are considered examples of a good teacher, mentor to everyone in the large family that is the organization. The leadership style is characterized by promoting teamwork, consensus and participation. The values that people share are loyalty, commitment to the organization and mutual trust. Teamwork is very important (Troilo, 2021). However, rapid transitions in digital technologies have introduced the concept of digital disruption. Digital distortion; It is the changes that occur in the basic attitude, behavior and culture of an organization caused by digitalization (Gartner, 2020). All these concepts have had profound effects on paradigm shifts. In this respect, the correct determination of the digital transformation process, in which the paradigm break is felt most deeply, has the power to close the strategic gap. The new paradigm is shifting from independence to interdependence, from control to connectivity, from competition to cooperation, from individual to group, and from tightly bound geopolitical alliances to more loosely connected networks. It turns out that there is a need for new leaders who can respond effectively to such conditions (Burn and Houston, 2015). In this respect, it should be understood what the guidance style suitable for the new era is. The current digital age is characterized by disruption. Digital leadership, generally defined as the appropriate leadership approach in the age of digital disruption, is defined by a company’s ability to support the affordable use of its assets at both the corporate and individual level to achieve its digital business goals (Meffert and Swaminathan, 2018; Breuer and Szillat, 2019). At this point, digital leaders play a critical role in the transformation of business strategies. It is necessary to be open to digital transformation and to use appropriate resources to facilitate this transformation (Bughin et al., 2020). However, although there are desired commitments in many enterprises, there has been a problem with adaptation to change. To prevent these issues, it is necessary for every organization to correctly recognize the eco-system it is in and to develop appropriate guidance, that is, a leadership style. In this respect, it has been revealed that there is an important relationship between business strategy and leadership through the concept of the innovative vigilance coefficient, especially in the studies conducted on the survival and development of organizations (Xiong, 2020). The basis of leadership is to create a unity of purpose and vision in the employees so that the business can achieve the desired strategic goals (Northouse, 2010). Of course, the important thing here is to understand the context and the period. From this point of view, in the context of the current period, Sow and Aborbie (2018) stated that when they researched the leadership styles that affect the digital transformation of an organization, the leadership style has a significant impact on organizational transformation and plays a critical role in the success of the change effort. Purpose of the research; is to reveal the leadership roles suitable for the digital transformation era. At this point, the hypothesis of the research is that there is a positive relationship between the success of digital transformation and digital leadership roles. In this respect, the study emphasizes that the digital role of the leaders of the age should be understood in order to direct the changing psychology of the organizations and adapt the business strategy to the new system. In addition, while introducing the new system that must be followed with the benefits of digital transformation, the fundamentals of digital leadership conceptually were conveyed as literature knowledge to understand the role of the digital leader in this system. In the methodology part of the research, the qualitative method was preferred and the conceptual relationship density was conveyed in the light of the findings obtained through content analysis.

## Requirements for digital transformation

The concepts of digital transformation or digitalization are understood as changes in society resulting from technological development (Chew et al., 2013). The benefits of digital transformation offer an opportunity for innovation in organizations. These opportunities range from how the business operates, the services it provides, the technology platforms it uses, how the business is organized, and where employees work. However, the dynamic and unpredictable market conditions brought about by digital transformation increase the risks associated with taking wrong strategic decisions and cause some changes in the firm’s strategy and business model. The lasting effects of the disruption that characterizes transformation mean that at a time when the relentless pace of digital innovation creates so much uncertainty, serious problems arise in terms of guiding and managing the strategic transformation of companies (Henry, 2013; Oliver, 2018). Digital transformation drives industrial life with both positive and negative benefits. The first thing to understand here is what the digital growth of technology means. Digital technology is conceptually defined as a process that combines information, computing, communication, and connectivity technologies to improve the performance of an asset by triggering significant changes in its properties (Vial, 2019). So, for a business, the digital transformation includes understanding the relevant connection points and positioning itself according to the new order in a way that will respond to the technological speed by it (Guinan et al., 2019). To be able to transform digitally; refers to the strategic adoption of new digital technologies and tools (Citrix Systems, Inc., 2020) to improve business processes and productivity and deliver better customer service and employee experiences. From the general perspective, the important and difficult thing in digital transformation is to find or apply the right technology (Oberer and Ve Erkollar, 2018). But despite the abundance of digital technology, not all organizations achieve the expected results (Troilo, 2021). At this point, having a business mentality that is open to and suitable for transformation comes to the fore before the conveniences brought by digital technology. Of course, it is necessary to analyze and identify the stages necessary for digital transformation. At this point (Zaoui and Souissi, 2020), making evaluations, creating a strategy and vision, drawing a roadmap, integrating customer experiences into the process, initiating operational transformation, designing digital transformation, producing strategic guidelines, implementing field implementation, providing corporate transformation, product and transforming the service offer, allocating the digital culture, creating a new business model that will create value, ensuring the integration of information technologies and preparing the structural harmony. An abundance of mind that is open to innovation is necessary for the benefits of technological abundance to transform into action. Now that you know what digital transformation is, you can define what the real challenge is. The main challenge in organizations is not just applying the right technology, but culture and skills. Because the general attitude and perspective of the organization are more important than technology (Rogers, 2016). In other words, if there is no culture and vision suitable for transformation, technology advantage alone does not work. Technology can be a car, but it takes skill and clarity of mind to use it. It is clear that digital transformation brings organizational change. In other words, organizational change is necessary for digital transformation. This study aims to bring attention to the focus of change, especially through return and necessity, regarding the digital transformation process. Organizational change is explained by adapting. At the beginning of the important difficulties in ensuring digital adaptation, there is the establishment of digital culture and the elimination of skills shortages at the establishment stage. Digital technologies have an intense relationship not only in the field of information technology but also with how businesses are managed (Oberer and Ve Erkollar, 2018). Kane et al. (2017) in their research; in an organization’s adaptation to an increasingly digital environment, one step before applying digital technology, the company’s strategy has drawn attention to the fact that the company’s workforce and corporate culture should be compatible with digital expectations. In the final analysis, the change in organizational processes seems to be both a return and a necessity of digital transformation. Indeed, Hinds (2019) highlighted digital transformation as a radical and continuous change in how an organization uses technology, people, and processes to fundamentally change the customer experience and business performance. Long and Lung (2002) investigated the interaction between technological change and organizational change and found that to create perfect harmony in technological change, the organization must undergo process change in many aspects. The purpose of this research is not only to decide. For this reason, it aims to provide a perspective on how change can be achieved. In explaining this purpose, we would like to make use of an analogy. Change is a long road, and technology is the car that makes progress on this long road. The question to be asked at this point is; who drives the car on the way to change?

## Digital leadership and its fundamentals

**Figure 1:**
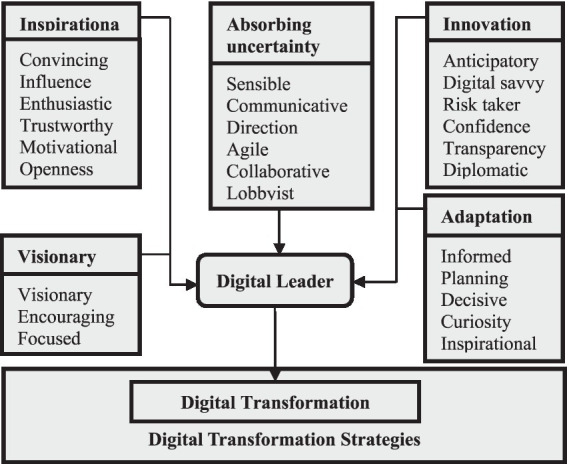
Digital leadership conceptual framework (Magesa and Jonathan, 2021).

Digital leadership carries many of the general leadership characteristics, it has a multidimensional structure that includes elements of transformational leadership, transactional leadership, and authentic leadership (Prince, 2018). Transformational leadership transforms followers and encourages them to think about the interests of the organization rather than their interests, motivates them, creates a vision, and encourages them to do their best (Bass, 1999). Transformational leaders are forward-looking and proactive in shaping the future of their organizations (Wu and Wang, 2015) Determine the need for change, and establish organizations and teams. It is also what determines the course of important changes such as digital transformation (Kark et al., 2018). The transactional leader is; It is based on traditional exchange relations or processes. It encourages the reward system to ensure employee obedience and thus creates a style that emphasizes the interaction between leaders and subordinates (Avolio and Ve Bass, 2013). But this style also includes punishing an employee who performs poorly or falls short of the target. In short, this leadership style enables the achievement of organizational goals by regulating the behavior of subordinates as desired or by preventing undesirable behaviors (Adriansyah et al., 2020). Authentic leadership, on the other hand, fosters a sense of togetherness in transformation and interaction. Digital leadership developed based on these three leaderships reveals that in addition to these features, it is necessary to develop a digital mindset and skill set to inspire and help corporate employees adapt and adopt digital changes (Magesa and Jonathan, 2021). At this point, one of the main functions of the leader; the task of reconciling individual values with organizational values (Bass, 1999) plays a key role. But to optimize digital technology that will create value, it is necessary to integrate culture and competence first. Therefore, these transformations promise success under a good digital leader (Qureshi, 2019). In this respect, digital leaders are a critical part of guiding the right transformation of companies with digital capabilities (Oberer and Ve Erkollar, 2018). Kane et al. (2015) draw attention to the roles that distinguish digital leaders from other leadership types and argue that there should be a clear digital strategy combined with leadership and culture ready to lead transformation. At this point, leaders trying to strengthen a digital culture; Their commitment to a new working approach and their tasks such as communicating entrepreneurial processes and ensuring progress by updating goals stand out (Guinan et al., 2019) As a matter of fact, Zupancic et al.’s (2017) studies show digital leadership competencies; human resources, architectural design process, digital ecologies, and collaboration environment. From this point, digital leadership is characterized by the use of an organization’s digital resources to achieve organizational goals and objectives (Shah and Patki, 2020). Digital leadership involves demonstrating agile leadership capabilities that enable the organization to create a culture of continuous innovation by using the latest technologies in business architecture (Tanniru, 2018). Digital leadership, defined as a leadership approach in line with the technological age identified with disruption (Meffert and Swaminathan, 2018), supports the calculated use of a company’s assets to achieve its digital business goals and is defined by its ability to address it at both the corporate and individual level (Breuer and Szillat, 2019). On the other hand, Larjovuori et al. (2016) according to; A digital leader is a guide with the ability to formulate a clear and meaningful vision and execute strategies for the digitalization process. The Figure 1 below presents a conceptual framework for digital leadership. The roles described within this conceptual framework were also used to justify the research design.

In the final analysis, digital leadership proposed by this study; builds the context (time, space) that harmonizes its business and strategy by changing conditions, supports a unifying culture that can integrate financial transformation into it, and has a visionary successful social, and intelligence in directing the emotions of employees, is open to new ideas, nurtures creative and design thinking, but maintains its charisma remotely and silently. It is reflective.

## The role of digital leadership in digital transformation: Benefit, necessity, and diffusion

The main question of the research is to focus on the role of the digital leader in the digital transformation process. While putting this forward, it is desired to draw attention to the features that are suitable for the business strategy required by the age. The research question aim is to reveal findings about both the role of digital leadership and its contribution to businesses. However, it is impossible to provide separate prescriptions for each industry and line of business. It may be more effective to focus on the personal character of digital transformation rather than looking at sectors. Considering all these aspects, and the enormous amount and speed of change, the character of digital transformation can be described as the VUCA environment from US-US military jargon. Volatility = frequent and strong changes. Uncertainty = unclear situations, lack of predictability. Complexity = interdependence of multiple elements, cause-and-effect chain unclear. Ambiguity = inconsistent and contradictory environment, cause-and-effect confusion. In such an environment, detailed analysis and planning becomes extremely difficult as business developments are often unpredictable. Therefore, some successful leadership approaches of the past are useless (Petry, 2018). Research from this perspective seems more likely to offer a valid prescription for digital leadership. Finding or implementing the right technology seems to be important and difficult for digital transformation, but this is not a stand-alone skill. At the beginning of the important difficulties in ensuring digital adaptation, there is the establishment of digital culture and the elimination of skills shortages at the establishment stage. At this point, digital technologies have an intense relationship not only in the field of information technology but also with how businesses are managed and what kind of leadership styles are applied (Oberer and Ve Erkollar, 2018). Today, digitalization leads to the transformation of production, logistics, communication, and human resource management. However, there is an important confusing point. It is necessary to correct the false perception that only technology-oriented businesses need digital leadership competencies. Because digital leadership competencies represent a wider area than the business line companies are affiliated with. At this point, it is necessary to mention the two most prominent key roles of digital leadership. Leaders trying to strengthen a digital culture first know how to communicate a commitment to a new work approach to employees and support entrepreneurial processes. Second, these leaders are a guide on how to monitor progress for updated business objectives (Guinan et al., 2019). The literature on digital transformation highlights changes in an organization’s leadership structure as an important enabler of new business models. To this end, the literature highlights the creation of new leadership roles (Vial, 2019). At this point, regardless of the sector, every business that wants to compete in today’s conditions will need digital leadership skills. However, to make this claim, it is necessary to know the nature of digital transformation. Digital transformation is a kind of development. This development has three interrelated perspectives. These are; economic, social, and human factors. This tripartite perspective is based on the fact that individuals are agents of change and manpower is key in the use of technology to achieve development results. From this point of view, businesses that want to digitally transform their services and operations need a leader to lead the change in a way that can provide this (Qureshi, 2019). Change management initiative requires effective leadership and, the leader acts as a pioneer to implement this change in the organization and makes the organization ready by making the necessary arrangements within the framework of existing business models. In the era of digital transformation, the leader tries to solve the digital impact and close the gap in the workforce by developing platforms and strategies based not only on the new digital platform but also on new business models. Because the competitiveness of digital transformation is basically about innovation (Al-Ali et al., 2017; Oberer and Ve Erkollar, 2018). At this point, digital leaders play a critical role in harmonizing business strategies to achieve an optimal balance suitable for the transformation of business strategies in the digital transformation process. Developing strategies suitable for digital transformation is demonstrated by being open to digital transformation and using appropriate resources to facilitate digital transformation (Bughin et al., 2020). The digital leader is fast, hierarchical, team-oriented, and collaborative, with a strong focus on innovation. This is the leader’s vision and foresight to digitally transform the organization.

Today, leaders need to know their digital skills and their impact on business, and how to leverage new technologies. However, this does not mean that they have to know how the technology works, it is important to know why it is important and how to use it (Özman et al., 2020). In this respect, digital leadership does not mean being the best programmer but being able to form a well-founded judgment about the opportunities and risks arising from technical progress (Liebermeister, 2019). In support of this, Anghel (2019) argues that digital transformation is more about leaders, managers, and employees’ perceptions of purchasing the change process and integrating the organization’s systems with new digital technologies, rather than technical expertise. Here, it is important not only to act with foresight and adapt to innovation but also to be the pioneer of innovation and to manage time effectively as a demander of innovation. At this point, the motto is “Early bird gets ahead.” In this respect, leadership is seen as a critical factor in supporting change management processes in an organization so that business strategies can achieve an optimal balance suitable for transformation in the digital transformation process (Michaelis et al., 2009). About the time management role of digital leadership, Willcocks (2022) argues for the cost of digital technology and argues for early investment in capabilities and technological platforming for digital transformation (Magesa and Jonathan, 2021). It is claimed that a digital leader has the role of inspiring, being visionary, managing uncertainty, being innovative, and adapting during digital transformation. As an upper reflection of these roles, the digital leader assumes a role that ensures the digital maturity of an organization with its digital vision and strategy, and places people, technology offers, and business models as a basis for participation in the process (Özman et al., 2020). Based on this role, another motto of digital leadership has been “strength from unity.” Overall, Digitization has changed the way organizations operate, but has never transformed an organization on its own. What helps an organization achieve such transformation or change is its leaders’ vision for digitization.

MIT Sloan Management Review conducted a study with senior executives. The findings of the research show that businesses need leaders who support a culture of change and lead the organization to digitally re-evaluate its business. Digital leader intelligence has come to the fore not only as a master in technology but also as a key element for the future of organizations (Kane et al., 2019). Westerman et al. (2014) focused on the role of the leader in realizing digital transformation and defined the digital leader as “the individual who mobilizes the organization through his digital awareness and power of influence.” At the same time, they talked about the necessity of behaviors that can be managed by digital leaders for the digital transformation of the organization. It seems that “emotional attention” is required in determining how to better reorganize their plans as existing internal and external conditions change. The importance of creating the right culture in offering the right products and services in coping with the competitive environment was emphasized.

In countries such as India, Japan, Australia, Brazil, China, the European Union, and the United States, a study was conducted to understand how digital technologies can be leveraged by business leaders and policymakers to accelerate business growth and competitiveness. According to the results of this research, nearly half of the 925 participant leaders found that the lack of digital skills is a critical factor for the transformation in the digital transformation process, and despite the size of the companies and business lines within the scope of the research, businesses are not ready enough for digital transformation (Özman et al., 2020). Another result of the research is that, regardless of the size of the companies, the required effect has not yet been awakened in the awareness level of the management. As it is known, organizational change is usually initiated by top management. First of all, they need to accept the necessity of change for themselves and then for the entire organization and persuade the organization to this change. However, it is not easy to persuade neither the managers nor the employees. In this respect, the integration of the organization into the transformation highlights a more important role than economic size, that is, having digital opportunities. Economic wealth can enable to establish the of digital infrastructure. But to optimize digital technology that will create value, it is necessary to integrate culture and competence first. Therefore, these transformations promise success under good digital leaders (Qureshi, 2019). At this point, it is extremely vital to reveal the role of digital leadership in harmonizing business strategies in digital transformation. The digital leader supports the transformation process of a company. It also has the power to coordinate and prioritize the transformation efforts of a pre-digital organization and reach the desired future state for strategic goals (Matt et al., 2015). Also (Khaw et al., 2022) highlighted the impact of digital leadership on a firm’s sustainable performance. In summary, the digital leader is essential for a firm’s digital technological success (El Sawy et al., 2016). But according to an MIT study, only 12% of the more than 4,000 organizations surveyed think their leaders have the mindset to drive digital change. Also, 82% believe leaders need digital skills to cope with the new competitive environment, while only 10% agree that leaders in their organizations have them (Ready et al., 2020). Currently, the dynamic and unpredictable market conditions brought about by digital transformation increase the risks associated with making wrong strategic decisions, which in turn causes changes in company strategy and business model. These effects of digital disruption pose serious difficulties for companies in guiding strategic transformation (Henry, 2013; Oliver, 2018). As companies seek their place in such a turbulent business environment, strong leaders are needed at the helm (Kane et al., 2019).

## Methodology

In this study, the qualitative method was used. The qualitative method is “an appropriate method to explore people’s experiences and practices, their perceptions and understanding of the research topic, and to explore sensitive issues (Braun et al., 2017). The focus of data analysis was initially to identify participants’ perceptions of digital leadership approaches in their organizations. The aim is to understand what the participants’ knowledge about digital leadership conceptually corresponds to. This stage was determined by phenomenological analysis. The phenomenological method mostly involves collecting data through a clinical interview. In this analysis, it is possible to collect individuals’ experiences or thoughts about concepts through group interviews and answers to open-ended questions (Marton, 1994). Nvivo software was used to obtain the findings of this research. This is because; this software provides advanced tools to visualize data, allowing deep analysis (Patton, 2002). This software can analyze, classify and categorize data from interviews (Welsh, 2002). In this research, it is important to make a phenomenological analysis first, to correctly determine the digital leadership roles, which is the main purpose of the research. At this point, while designing the research; we used the McKinsey: Digital/McKinsey (2018) report. According to this report; The industry analysis shows that less than 30% of digital transformation programs are successful, further revealing that “having the right, digitally savvy leaders in place” is one of the five success factor categories to drive digital transformation success. As reported in the literature, according to an MIT study, only 12% of the more than 4,000 organizations surveyed think their leaders have the mindset to drive digital change. Also, 82% believe leaders need digital skills to cope with the new competitive landscape, while only 10% agree that leaders in their organizations have them (Ready et al., 2020). This information has provided us with inspiration and rationale for determining digital leadership roles. In addition, different bibliometric analysis contents for research design (Petry, 2018; Marvasti, 2019; Vial, 2019; Khaw et al., 2022) were examined and included in the justification. Content analysis was used to determine digital leadership roles. The content analysis describes the reduction of textual data into measurable segments after coding. It includes outlining the problem, obtaining the material, determining the focus, and counting the occurrences of the categories (Marvasti, 2019). On the other hand, it systematically explains the meaning of qualitative material made by classifying the material as examples of coding categories (Schreier, 2012). The purpose of using the MAXQDA 20 program is to understand the relationship analysis; In sampling, focus group interviews (Kitzinger, 1995) are preferred because of their systematic structure, which provides the opportunity to establish a solid basis with detailed one-on-one interviews with data. In this program; Recorded interviews are transcribed verbatim to analyze data from focus groups. It then allows codes to be made with the MAXQDA software program (MAXQDA, 2020). The last reason is; that this program helps to provide intuitive access to the statements and contributions of selected participants of the focus group (Kuckartz and Radiker, 2019). MAXQDA provides functions specifically tailored for qualitative data analysis of focus group data (Saillard, 2011). In this research, 34 different leaders working in leading real sector organizations were interviewed. Semi-structured interviews consisting of open-ended questions were conducted through digital platforms due to pandemic measures. At this stage, approximately 35 pages of data were collected. The main research question that shaped the discussion in this article was: What is the role of digital leadership in helping businesses achieve strategic alignment in the digital transformation process? Why is it important to understand these roles?

## Findings

The facts that are important in explaining digital leadership have been previously studied in the literature. At this point, the concepts repeated by the leaders must overlap with the literature and have similar meanings to these concepts. Nvivo 12 package was used for data analysis of the information provided by the leaders in the obtained documents. The words, sentences, and paragraphs in the interview texts are separated according to their usage and frequency. According to this; while explaining digital leadership, the concepts that are given priority and used together, “adaptation and system effectiveness,” and “change” about this came to the fore. These concepts are highlighted in the effectiveness of a business. In addition, “productivity with time management” and “costing” about this were highlighted. Clustering analysis according to the word, which is the type of evaluation obtained in the analyzes made with Nvivo, is shown in Supplementary Figure S1.

The importance of similarity in this analysis; is to provide information about the level of conceptual knowledge of the participants in explaining the subject. The facts that are important in explaining digital leadership have been previously studied in the literature. Obtaining results parallel to the literature has been a supportive element in terms of contextual examination of the relationship between concepts. Another data obtained with the NVivo program is the single direct weight relationship. According to this; While the participants are explaining the researched concept, the weight of which concept is used as the main concept with other concepts is revealed. The information on this map is also a confirmation of the findings presented in Supplementary Figure S1. There is a direct proportionality between the size of the circles and the load of the weight in this map, which helps to explain the conceptual relationship weight of Digital Leadership.

The finding in Supplementary Figure S2 shows that digital leadership is conceptually conceptualized with system effectiveness in the digital transformation process, and efficiency, adaptation, and time management come to the fore in the direct-weighted relationship of this concept. In addition, the weight between “adaptation” and “change” and “time management and productivity” confirmed the findings in Supplementary Figure S1. Another data obtained with the NVivo program is the Coverage density relationship. According to this; It is revealed which concept used by the participants in the research area covers the other concepts used. The information in this map is also a confirmation of the findings in Supplementary Figures S1, S2.

Another data obtained with the Nvivo program is that the finding in Supplementary Figure S3 is that the phenomenon concepts revealed in the explanation of digital leadership are explained by which other concepts. According to the findings; The concepts of productivity, employment, and costing are explained within the scope of time management. Another finding, system efficiency, came to the fore with adaptation and efficiency, and it was observed that the change was explained within the scope of adaptation. These findings also confirmed the findings in Supplementary Figures S1, S2. Content analysis was carried out in the next phase of the research. The content analysis stage is the stage in which the answer to the research question is sought. At this stage, the MAXQDA 20 package was used for data analysis. In this program, the data is coded with the program interface, separated into themes, and made ready for interpretation. To determine the relationship between the codes given through the MAXQDA 20 program, the frequency of the codes used together in the same sentence or paragraph was analyzed. As a result of the analysis, the matrix in Supplementary Figure S4 was obtained. The interaction between the code relations scanner and different codes has been quantitatively transferred. The aim is to interpret the relationship between the codes qualitatively and quantitatively. The words, sentences, and paragraphs in the interview texts were interpreted and coded. As a result of the coding process, 8 main codes were determined.

According to Supplementary Figure S4; the relationship between the codes was analyzed according to the coding frequency in the same sentence or paragraph. According to this analysis, the number of interactions between various codes was evaluated quantitatively. The purpose of the evaluation is to show the strong and weak correlations between the codes. Elements used by the research participants in their evaluations. According to this matrix; “System efficiency” 32 times, “Time Management” 31 times, “Adaptation” 28 times, “Productivity” 19 times “Costing” 17 times, “Change” 15 times, “Effect” 9 times, and “Employment” “It was used together 8 times and the relationship was transferred. The relationships revealed by the code relations scanner provide information about the roles of digital leadership and the intensity of the benefits it will provide to businesses at some point. The most special result in this matrix is the digital leadership’s relationship between time management and productivity, and the most intense data on this relationship is the return it will provide to companies in terms of cost. In addition, by associating the system adaptation of a well-managed time with change, the benefit of digital leadership comes to the fore in making the change promptly. All this pool of relationships highlighted the role of digital leadership in system effectiveness. Another data obtained with the MAXQDA program; has been code-based frequency analysis and is transferred to Supplementary Figure S5.

Code-based frequency analysis; is to show how much it emphasizes each code assigned to the answers quoted in the answer text. Looking at the data obtained from the research, it was determined that 7 codes were concentrated at different rates. 100% of the participants included in the research were “Adaptation,” 92.3% “System efficiency,” 92.3% “Change,” 76.9% “Costing,” 69% It is seen that, 2 of them focus on “Productivity,” 53.8% on “Employment” code. The most important feature of this finding; is to reveal the key role in the work done by making use of the focus group experiences in which the interviews were conducted. Another data obtained with the MAXQDA program; is a single case model. In this model, all data is in a single structure. It differs from code-based frequency analysis in that it gathers each participant in this single structure. The model is evaluated as a single source of the obtained response texts and a uniform code relationship is revealed. In this context, while coding, all of the assigned codes were evaluated as if they were obtained from a single text, and the group’s weighted common point of view was revealed. While code-based frequency analysis evaluates each document one by one, the single case model is the evaluation of all documents. The single case model is illustrated in Supplementary Figure S6.

In this model, the thickness of the lines coming out of the single structure containing the interview texts transferred to the codes shows the intensity of the relationship between the focus group and the relevant code. The quantitative density of the lines coming out of the codes provides the model by detailing the population density. Findings from the model It has been determined that Digital leadership, which is put forward to prevent businesses from experiencing strategic deficit problems in digital transformation, is in the role of providing organizational adaptation, time management, and system efficiency, or it benefits the business strategy in this respect. Another data obtained with the MAXQDA program; is a word cloud. According to this type of find; It is the creation of a common pool of the most frequently used words while demonstrating the digital leadership roles of the participants in the digital transformation process. If the words in this pool are larger or bolder than the other, it means that the word is emphasized more by the managers.

The interpretation of this type of finding in qualitative research seems similar, but it is confused with the single case model in Supplementary Figure S6 in terms of interpretation according to thickness with the code-based frequency analysis transferred in Supplementary Figure S5. However, it should not be forgotten that the words quoted in Supplementary Figure S7 characterize the sentences directly formed by the participants participating in the relevant research. On the other hand, in Supplementary Figures S5, S6, what is the main idea as latent information in the contents obtained from the discourses of the participants? Based on the question, the quality of the codes we make as experts is at the forefront. When we look at the word pool used in Supplementary Figure S7, it has been determined that the participants have the conceptual power to reveal qualifications about the scope of digital leadership.

## Conclusion

In this study, we tried to put forward a concept. In the first place, we explained the structural and organizational forms of digital transformation. Then, we gave information about the basic characteristics of the guiding character, that is, the leader. In this study, which is based on the philosophy that it is not very possible to present a leadership concept suitable for every business and sector in the last stage of the literature; We aimed to convey the leadership roles that can fit the structure of digital transformation, which is characterized by disruption, and how these roles can contribute to the business. We conducted the research with two qualitative analyzes. This has two purposes. The first of these is the effort to bring qualified workers to the literature. The second is to understand the capacity of the participants to perceive and recognize the variable we are working with due to the specific aspect of digital transformation. Why is this important? Because qualitative studies reveal the characteristics of a particular group. The conceptual lack of that group in the field of work can weaken the work in terms of content and can lead to unsuccessful and meaningless results. We called this the qualification verification of the study. According to the results obtained from the study; the leaders who participated in the research correctly perceived and understood the fundamentals of the digital leadership phenomenon. The results of the study produced parallel results with other research on digital leadership but also gave different information from other studies. For example; Practical results emerged that companies should adapt to change, and use time effectively at this point, and this will ensure the effectiveness of the system in the name of efficiency. When these results are evaluated deeply, the relationship of the digital leader, especially time management and productivity, not only supports organizational adaptation but also characterizes being the initiator and pioneer of the transformation itself. Being the initiator of transformation is an inevitable aspect of organizational change. At this point, the practical result of the relationship between change and adaptation characterizes a feature that comprehends the features of digital technology and can explain it to its audience through cultural transfer. The intensity of the adaptation relationship with the effectiveness of the organizational system; characterizes the role of designing and integrating organizational strategy into technological development.

## Discussion

When the literature is examined; (Westerman et al., 2014; Kane et al., 2015; Sia et al., 2016; Kane et al., 2019; Mihadjo and Alamsjah, 2019; Özman et al., 2020; Magesa and Jonathan, 2021), it will be seen that researchers such as these point to similar results. However, the different side of this article is its feature of systematically presenting the results of studies that have been handled from many perspectives. The most specific and unique result of the study, which is not frequently encountered in other studies; is the role of the digital leader in managing operating costs. The scheduling role of the digital leader characterizes the role of saving on administrative expenses regarding the flexibility of the venue and platforms where the work will be done. It seems that; The presence of digital technology in the digital transformation process is not enough to direct the organization to the intended goals. The main challenge here is not just to apply the right technology, but also to culture and capabilities. The overall attitude and perspective of the organization are more important than digital technology. Therefore, making the digital leadership impact visible that will convey the culture and vision in the right direction is a key element for business strategies to achieve an optimal balance. The common misconception is that such information is already known. Then where is the problem? Years of research on conversions have shown that the success rates of these conversion efforts are consistently low (less than 30% successful). This shows that digital technologies are not easily realized (De la Boutetiere et al., 2018). According to an MIT study of international business leaders, only 12% of the more than 4,000 organizations surveyed think their leaders have the mindset to drive digital change. Also, 82% believe leaders need digital skills to cope with the new competitive environment, while only 10% agree that leaders in their organizations have them (Ready et al., 2020). In a different study conducted jointly in countries such as India, Japan, Australia, Brazil, China, the European Union, and the United States; Almost half of the 925 leaders found that the lack of digital skills is a critical factor for the transformation in the digital transformation process, and despite the size of the companies and business lines within the scope of the research, businesses are not ready enough for digital transformation (Özman et al., 2020). Research tells companies out loud that they need a digital router. So we have provided companies with a recipe for what the roles of these routers are, and what they should look for.

We also made a recommendation for companies; as it is known, organizational change is usually initiated by top management. Therefore, company management must first accept the necessity of change for themselves and then for the entire organization and convince the organization to change. However, it is not easy to persuade neither the managers nor the employees. To optimize digital technology that will create value, it is necessary to integrate culture and competence first. The first step of this integration is human resources. At this point, companies; it is recommended to find, select and place employees who are suitable for the corporate culture and digital culture from the very beginning. In addition, it is reminded that finding, selecting, and placing employees is a cost item that is not felt in the process but is large in the balance sheet.

## Research constraints and vision for future research

This research was conducted with leaders working in sectors such as aviation, health, tourism, energy and technology in Turkey in 2022. The place and time period of the study are two different contexts constraining this study. In addition, since a qualitative method was determined in this study, it has a different limitation in terms of generalizability to all sectors. It seems that digital transformation is not a boomerang that repeats itself and goes back to where it started. This cycle is not a vicious structure. For this reason, conducting new academic studies with different research techniques in different countries, regions and sectors, both in terms of the character of digital transformation and the leadership roles that will guide the transformation, will carry the transformation even further.

## Author’s note

Abdullah Türk received his bachelor’s degree from Anadolu University, Department of Business Management, his master’s degree in management organization and his doctorate from Beykent University in business management. He is currently working as an Assistant Professor in the Department of Aviation Management at Istanbul Bilgi University.

## Data availability statement

The original contributions presented in the study are included in the article/Supplementary material, further inquiries can be directed to the corresponding author.

## Ethics statement

Ethical review and approval was not required for the study on human participants in accordance with the local legislation and institutional requirements. The patients/participants provided their written informed consent to participate in this study.

## Author contributions

The author confirms being the sole contributor of this work and has approved it for publication.

## Conflict of interest

The author declares that the research was conducted in the absence of any commercial or financial relationships that could be construed as a potential conflict of interest.

## Publisher’s note

All claims expressed in this article are solely those of the authors and do not necessarily represent those of their affiliated organizations, or those of the publisher, the editors and the reviewers. Any product that may be evaluated in this article, or claim that may be made by its manufacturer, is not guaranteed or endorsed by the publisher.
