# Supplementary material for: Digital leadership role in developing business strategy suitable for digital transformation
Source: Front Psychol. 2023 Jan 4;13:1066180. doi: 10.3389/fpsyg.2022.1066180 (PMC9845252; doi:10.3389/fpsyg.2022.1066180)

**Supplementary Material**

**Supplementary Figure 2.** **Clustering Analysis by Word**


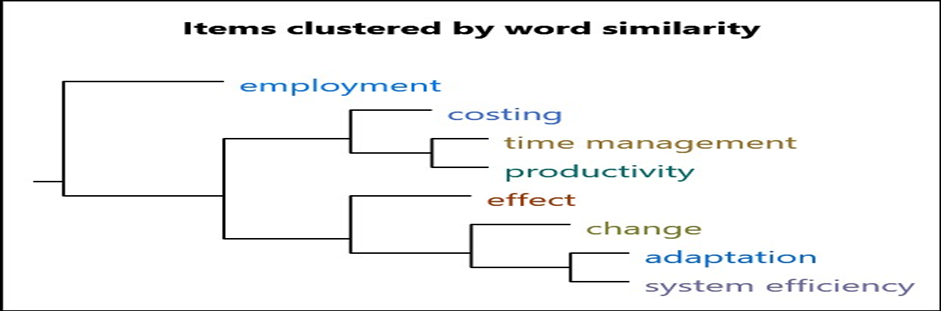


**Supplementary Figure 3.** Single Direct Weighted relationship


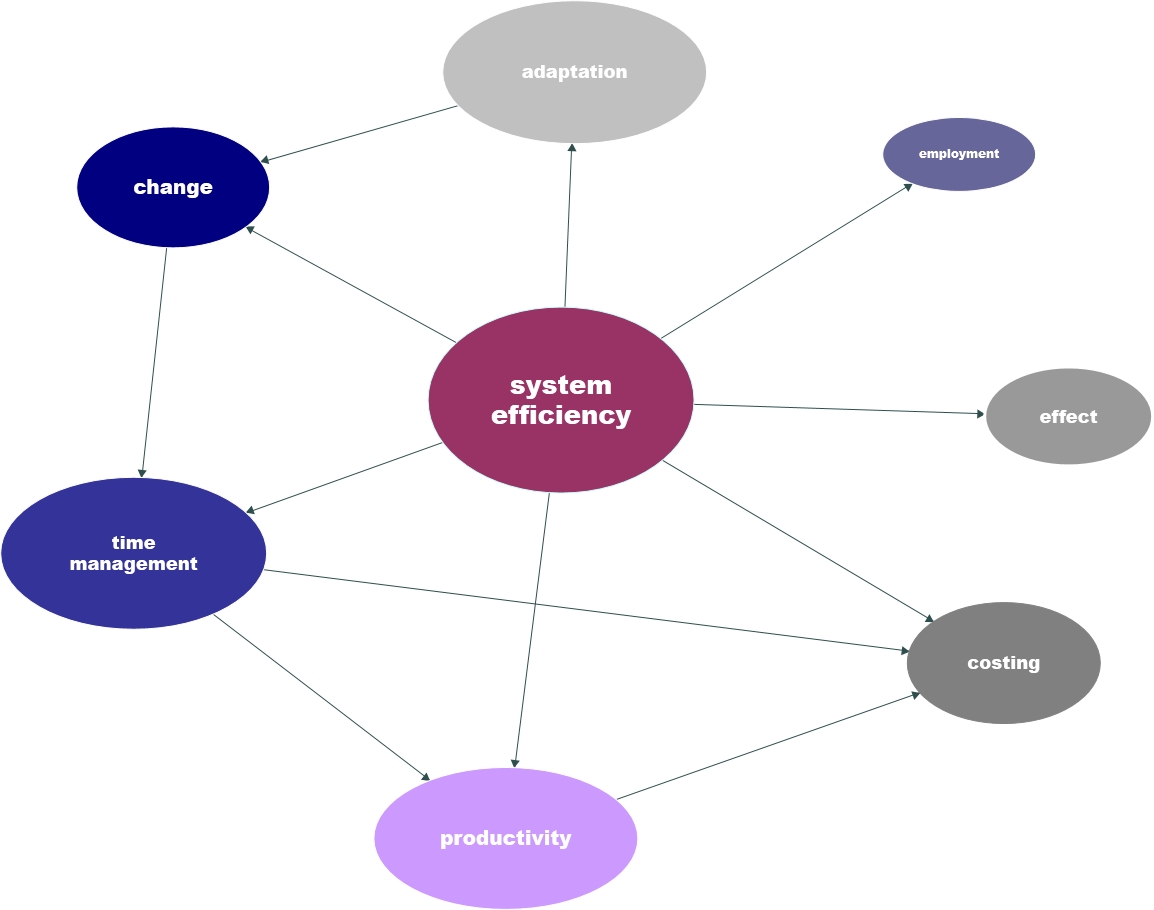


# Supplementary Figure 4. Scope Density Relationship


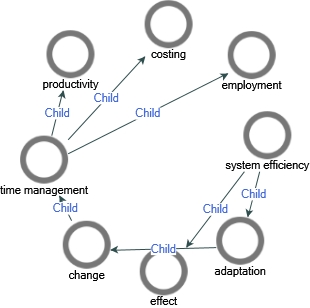


# Supplementary Figure 5. Code Relationships Browser


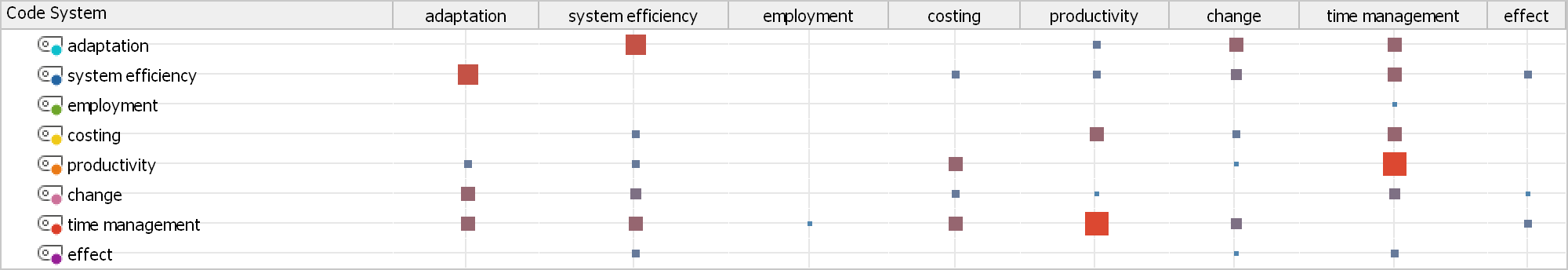


**Supplementary Figure 6.** Code Based Frequency Analysis


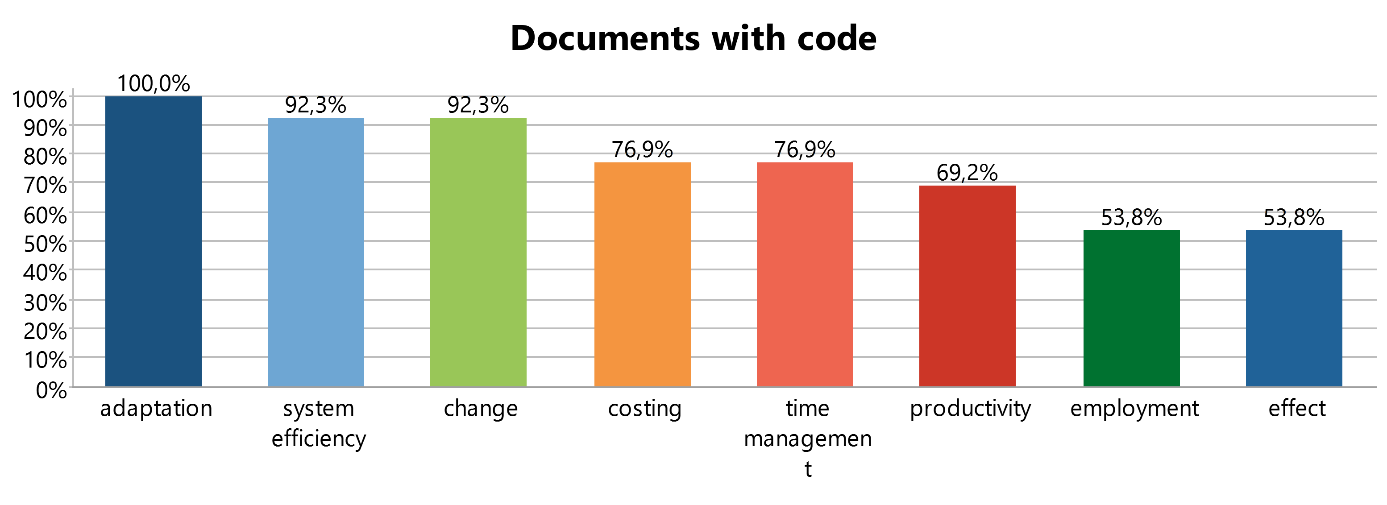


**Supplementary Figure 7.** Single case model


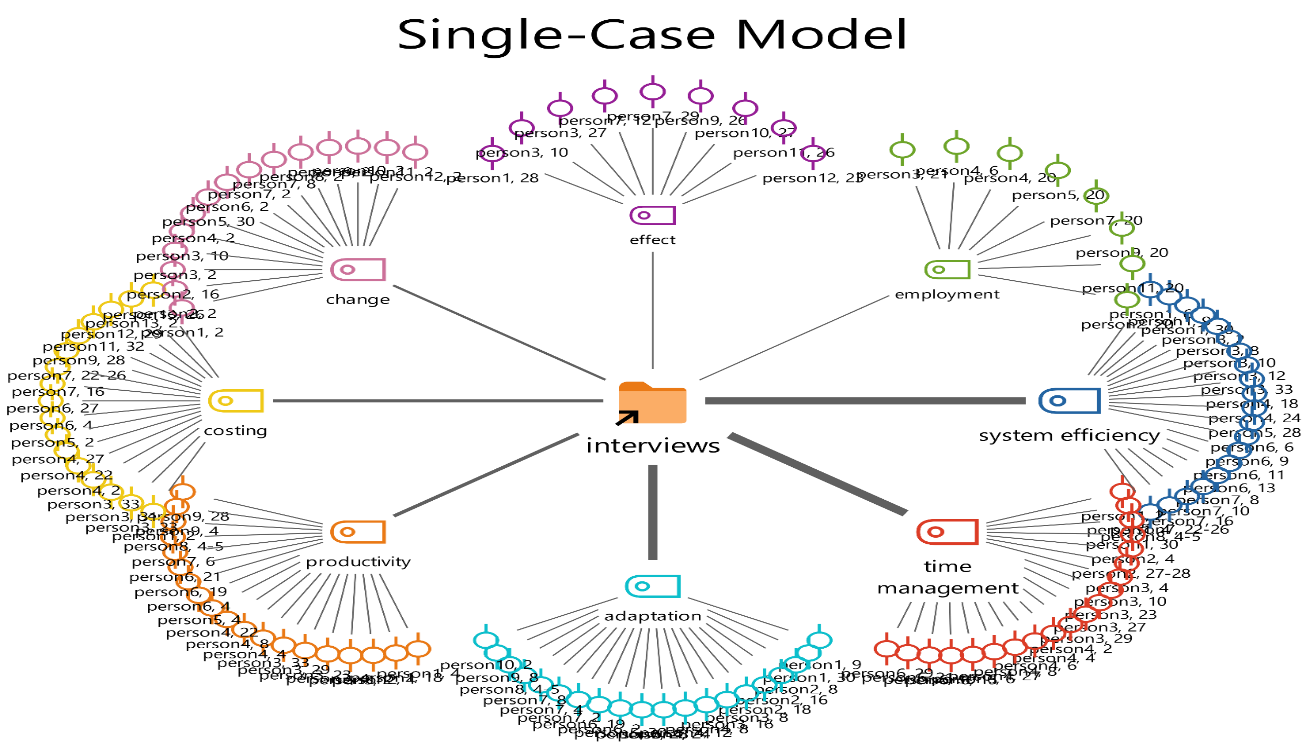


**Supplementary Figure 8.** Word Cloud


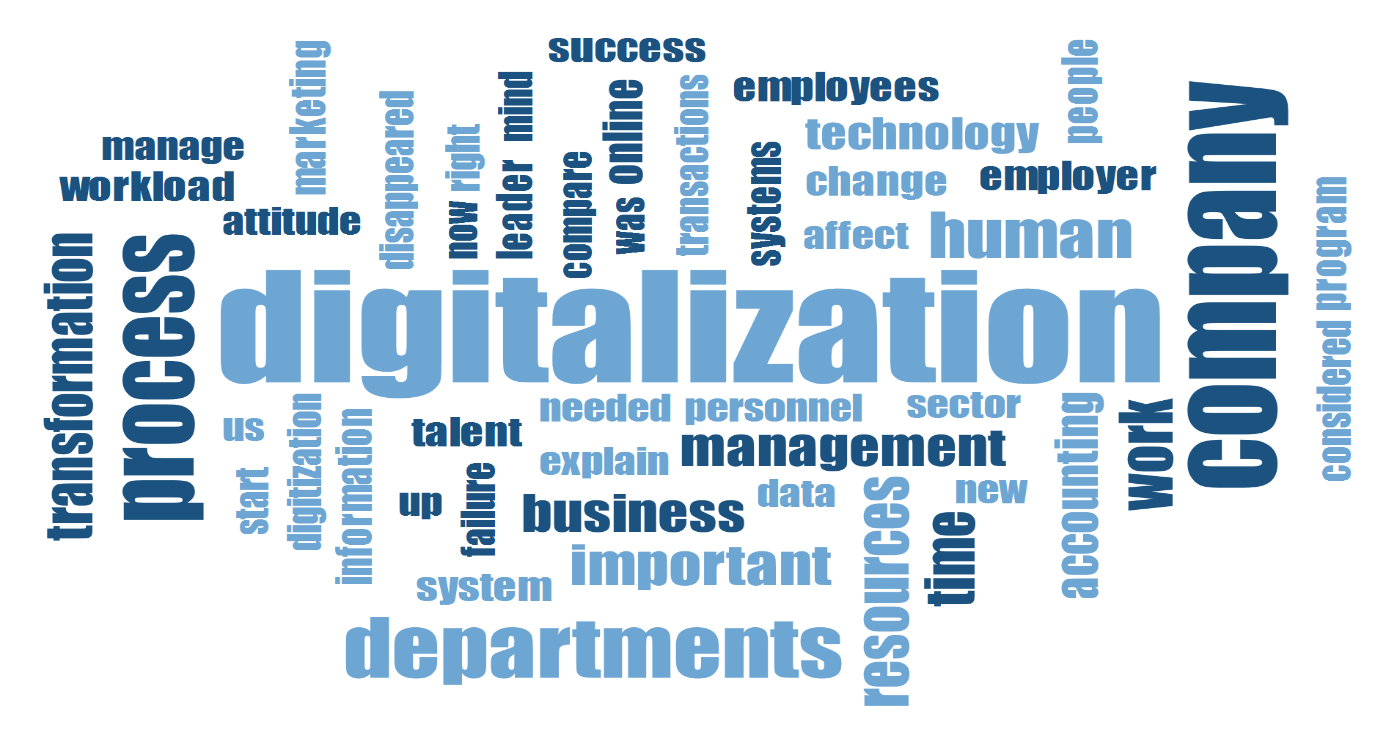

Supplement: Supplementary file 1 [file Data_Sheet_1.docx]
